# Supplementary material for: Prevalence and characterization of asymptomatic thyroid nodules in Assin North District, Ghana
Source: PLoS One. 2022 Feb 2;17(2):e0263365. doi: 10.1371/journal.pone.0263365 (PMC8809614; doi:10.1371/journal.pone.0263365)
Supplement: Attachment — Submitted filename: Response to Reviewers R2.pdf [file pone.0263365.s002.pdf]

The Academic Editor
PLOS ONE

Dear Sir

Prevalence and Characterization of Asymptomatic Thyroid Nodules in Assin North District, Ghana (PONE-D-20-33859)

Thank you for the review and providing comments to improve our manuscript. Below are answers or responses to specific comments made by the Editor and the reviewer.

Additional Editor Comments (if provided):

Please check the references. For instance, #27 was cited earlier as #21.

Author's response #1: Thank you for the observation. We revised our references. Reference #21 and #27 represented a single article, so we have reconciled.

Reviewers' comments:

Reviewer's Responses to Questions

Author's response #2: In line with the reviewers' responses to question 1 to 7, no further editing was required from the authors. However, we have thoroughly proofread the manuscript to correct any minor errors found. Please, see the revised manuscript with Track Changes

We believe our responses will be considered for onward publication

Thank you for your comments and inputs

Sincerely,

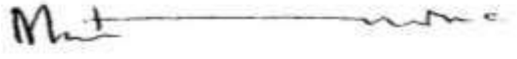

Dr. Martin T. Morna
