# Supplementary material for: Prevalence and characterization of asymptomatic thyroid nodules in Assin North District, Ghana
Source: PLoS One. 2022 Feb 2;17(2):e0263365. doi: 10.1371/journal.pone.0263365 (PMC8809614; doi:10.1371/journal.pone.0263365)
Supplement: Attachment — Submitted filename: Response to Reviewers.docx [file pone.0263365.s001.docx]

The Academic Editor
PLOS ONE

Dear Sir

**Prevalence and Characterization of Asymptomatic Thyroid Nodules in Assin North District, Ghana (PONE-D-20-33859)**

Thank you for the review and providing comments to improve our manuscript. Below are answers or responses to specific comments made by the reviewer.

Editor’s comments:

*1.) Please ensure that your manuscript meets PLOS ONE's style requirements, including those for file naming. The PLOS ONE style templates can be found at* <https://journals.plos.org/plosone/s/file?id=wjVg/PLOSOne_formatting_sample_main_body.pdf>

and

<https://journals.plos.org/plosone/s/file?id=ba62/PLOSOne_formatting_sample_title_authors_affiliations.pdf>

**Correction #1**

The format of the manuscript has revised according to the PLOSE ONE style

*2) Please amend your Ethics Statement to include information about informed patient consent provided for the study. Please state what type of consent was given (i.e., written, verbal, etc.). Additionally, please specify the type of consent in the Methods section of the manuscript.*

**Correction #2**

Both verbal and written consent statement added to ethics statement and in the Methods section of the manuscript

*3.) We note that you have indicated that data from this study are available upon request. PLOS only allows data to be available upon request if there are legal or ethical restrictions on sharing data publicly. For information on unacceptable data access restrictions, please see* [*http://journals.plos.org/plosone/s/data-availability#loc-unacceptable-data-access-restrictions*](http://journals.plos.org/plosone/s/data-availability#loc-unacceptable-data-access-restrictions)*.*

**Correction #3**

a. It is researchers’ own data and there are no ethical restrictions on sharing the data set of this work. Only that, we included the names patients and contact addresses from the community to conduct follow-ups. We will create a copy without the names and contact information and share the data set (anonymized data) with your on submission of revised manuscript.
b. We have shared anonymized data as indicated

**Response to major comments:**

*Introduction:*

*A description about the country or city in terms of previous estimates or importance of estimating thyroid nodule is required.*

**Answer #1:**

Thank you for your suggestions. We have revised the final paragraph of the introduction with further details on the significance of the study to our country or city and the relevant of estimating thyroid nodules in terms of epidemiological and clinical needs.

***Methods:*** *1. The authors mentioned that the household were selected using systematic random sampling. but sampling method is inadequate. Sampling frame should be clearly defined. How were the household defined and selected? What list did you select from?*

**Answer #2:**

Thank you for your observation and comment. We have revised this section in the manuscript with a clearer definition of what constituted a household in the current study as well as the sampling frame.

*2. One of the most important question in this study was the salt intake. However, there is no information regarding the items of this scale. in the result, there are just two answer (often, not often) for this question that is not enough. more description about the measurement of this question is required.*

**Answer #3:**

Thank you for your observation and comment. Your observation is genuine one and we have taken note of that in our study limitations, which should be considered in subsequent studies.

In this study, “often” intake of dietary salt was measured as daily consumption of >5g or one teaspoon of iodized salt, and “not often” intake of dietary salt was measured as daily consumption (<5g or less than one teaspoon of iodized salt or not at all).

All these updates have been made in the main write-up

*3. The statistical method for estimating the prevalence is not satisfactory. A more valid weighting estimate according to the population size of each community such as inverse probability weighting is required.*

**Answer #4:**

Thank you for your suggestions and comment. Weighting for dataset in estimating the prevalence of thyroid during analysis was not required as the design addressed sample weighting before data collection stage by taking into consideration sampling proportionate-to-population size in each community

*Results: According to the previous comment on calculation a weighted estimate of prevalence, the result need to be changed and corrected.*

**Answer #5:**

Thank you for your comment and suggestions. We duly acknowledge your genuine comments but we believe this has been duly addressed based on our initial responses in #4 above However, we have added the distribution of symptomatic thyroid nodules by community studied as Figure 1.

Minor comment:
Throughout, the language in the manuscript could benefit from editing by a native English speaker. The authors’ meaning is clear (nearly) everywhere, but the manuscript could benefit from a careful editorial review. Some words are unclear such as; contests, chin, multivariate level (instead of multivariate model), associating,…

**Answer #6:**

Thank you for your observations and suggestions. We have conducted thorough editing as suggested to make the write-up read better

We believe our responses will be considered for onward publication

Thank you for your comments and inputs

Sincerely,

Dr. Martin T. Morna
